# Latent depressive profiles and associated factors among overweight/obese individuals based on the socio-ecological model: a cross-sectional national survey in China

**DOI:** 10.3389/fpsyg.2025.1644701

**Published:** 2025-08-18

**Authors:** Xiaoping Yang, Miaomiao Chen, Xiaohui Liu, Lijun Wang, Yanyun Wang, Yingjie Zheng, Shailing Ma

**Affiliations:** ^1^General Hospital of Ningxia Medical University, Yinchuan, Ningxia, China; ^2^School of Nursing, Ningxia Medical University, Yinchuan, Ningxia, China

**Keywords:** body mass index, depression, depressive subtypes, multilevel factors, mental health

## Abstract

**Background:**

Overweight/obesity is associated with an increased risk of depression, which compromises the mental health of affected individuals. This study aimed to identify distinct depressive subtypes among overweight/obese individuals and examine associated multilevel factors based on the socio-ecological model (SEM), for guiding interventions enhancing mental health in this population.

**Methods:**

Data were derived from the Psychology and Behavior Investigation of Chinese Residents in 2021 (PBICR 2021). Assessment instruments included a General Information Questionnaire, the Patient Health Questionnaire-9, the Eating Behavior Scale-Short Form, the Family Health Scale-Short Form, and the Perceived Social Support Scale. Latent profile analysis (LPA) was employed to identify depressive subtypes, and multinomial logistic regression was used to examine associated multilevel factors across the identified subtypes. Analyses were conducted using SPSS 24.0 and Mplus 8.3.

**Results:**

This study included 2,588 participants classified into low-level (52.3%), moderate-level (36.6%), and high-level depression (11.1%) groups. Compared to the low-level group, high-level depression was significantly associated with age (18–45 years), current medication count (≥3, excl. supplements), out-of-pocket medical expenditures, higher abnormal eating behavior scores, and lower family health and social support scores. Similarly, moderate-level depression showed significant associations with female gender, age (18–45 years), having chronic conditions, current medication count (≥3, excl. supplements), out-of-pocket medical expenditures, higher abnormal eating behavior scores, and lower family health and social support scores.

**Conclusion:**

Depression demonstrates significant heterogeneity in overweight/obese individuals, with three distinct latent profiles identified. These findings highlight the need for future primary healthcare to prioritize personalized, depression subtype-specific interventions for overweight/obese individuals, guided by multidimensional factors identified through SEM, to improve mental health.

## 1 Introduction

Overweight and obesity are defined as abnormal or excessive fat accumulation posing health risks (Park et al., 2022). No country to date has successfully curbed the rising rates of adult overweight and obesity (Collaborators GBD 2021 Adult BMI, 2025). China currently has the world’s largest overweight/obesity epidemic, with 402 million affected adults (Collaborators GBD 2021 Adult BMI, 2025). Overweight/obesity is associated with increased risk and earlier onset of non-communicable diseases such as diabetes and cardiovascular diseases, as well as adverse psychosocial outcomes including low self-esteem and depression (Latzer and Stein, 2013). Substantial evidence confirms a significant association between overweight/obesity and depression (Cui et al., 2018; Gerardo et al., 2025). Compared with non-obese individuals, adults with obesity exhibit a 23–36% higher likelihood of developing depression and a 14–34% increased odds of major depressive disorder (Fulton et al., 2022). The harms caused by depression to overweight/obese individuals include impairment of personal well-being and quality of life, weakening their willingness to seek and adhere to treatment interventions, and the interaction between metabolic and emotional disorders can make despair, overeating and lack of exercise persist, thus forming a vicious cycle (Fulton et al., 2022).

To our knowledge, most studies assess depressive levels in overweight/obese individuals using depression scale scores or cutoff values. However, this “variable-centered” approach overlooks individual heterogeneity, resulting in findings that fail to accurately capture the actual depressive status of this population. Latent profile analysis (LPA) is a “person-centered” statistical method that identifies distinct latent subgroups within a population by analyzing individuals’ response patterns across multiple observed variables. Unlike traditional classification approaches that rely on fixed cutoffs (e.g., categorizing individuals as depressed or non-depressed), LPA offers a more nuanced understanding by grouping individuals who share similar symptom profiles (Chen et al., 2024). This approach provides more precise and objective classification results by capturing the underlying heterogeneity in population characteristics. LPA has been extensively utilized to identify depressive subtypes across diverse populations. For instance, Hou et al. identified three distinct depressive subtypes among older adults living alone using LPA (Hou and Zhang, 2023). Keins et al. (2021) demonstrated the existence of four clinically relevant depressive subtypes in patients with intracerebral hemorrhage through LPA. Nevertheless, the identification of depressive subgroups using LPA remains unexplored in overweight/obese populations.

**Figure 1:**
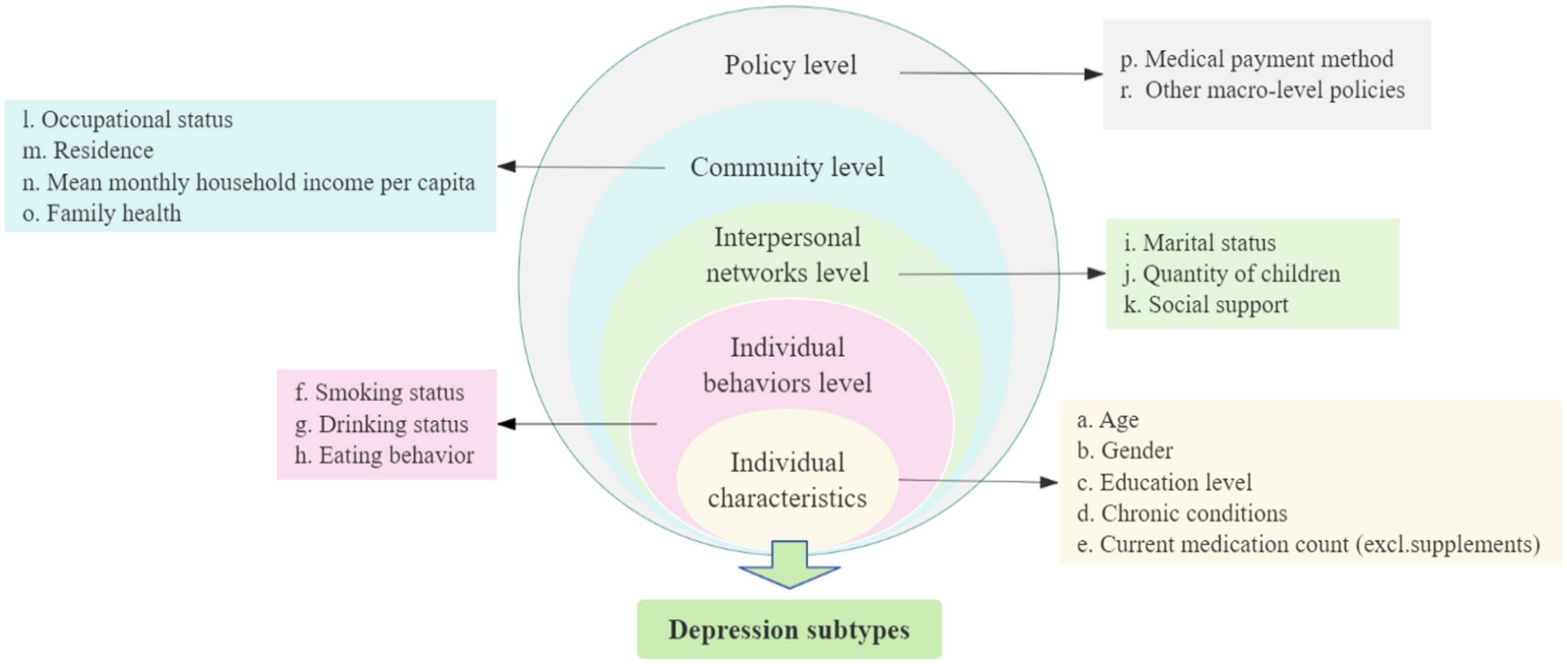
Factors associated with depressive subtype based on the socio-ecological model.

Additionally, previous studies on influencing factors of depression primarily focused on demographic characteristics and psychosocial factors, exhibiting a relatively narrow scope and lacking systematic exploration (Song et al., 2023; Bai et al., 2024). To address this limitation, the present study employs the social-ecological model (SEM) to identify depression-related factors in overweight/obese individuals (Figure 1). The SEM posits that individual health is jointly shaped by five interconnected levels: individual characteristics (intrinsic individual demographic, biological, and fundamental health characteristics), individual behaviors level (individual behavioral patterns and health-related behaviors), interpersonal networks level (social relationships and support systems), community level (living/working conditions and socioeconomic status), and public policy (local, state, and national laws and policies) (McLeroy et al., 1988). This multi-level framework facilitates comprehensive analysis of health determinants and supports the development of systematic health improvement strategies. The SEM has been widely used to examine health determinants across populations. For example, Jung et al. (2025) applied this model to analyze multilevel factors influencing future anxiety in Korean residents, while Huang and Tan (2024) employed a five-level socio-ecological framework to investigate cervical cancer screening participation among Singaporean women.

Therefore, this study aims to identify distinct depressive subtypes among overweight/obese individuals using LPA, and identify associated multilevel factors based on the SEM, to guide interventions that enhance mental health in this population.

## 2 Materials and methods

### 2.1 Survey design and participants

Our data were derived from a large-scale cross-sectional survey, the Psychology and Behavior Investigation of Chinese Residents in 2021 (PBICR 2021).^1^ This survey was conducted from July 10 to September 15, 2021. The project team surveyed using a multistage sampling method across 31 provinces/autonomous regions/municipalities in mainland China. Using the random number table method, 120 cities were selected, including the capital and two to six prefectural cities in each province/autonomous region. Based on data from the Seventh National Population Census of China (2021), quota sampling was applied to selected residents from these 120 cities (quota attributes: sex, age, and urban–rural distribution), ensuring the distributions of these variables in the final sample aligned with population characteristics.

According to the *Guidelines for the diagnosis and treatment of obesity in China* (National Health Commission of the People's Republic of China, Medical Administration Bureau, 2025) body mass index (BMI) remains the standard metric for defining overweight and obesity. BMI = weight (kg)/height (m)^2^, the criteria are: underweight (BMI < 18.5 kg/m^2^), normal weight (18.5 kg/m^2^ ≤ BMI < 24.0 kg/m^2^), overweight (24.0 kg/m^2^ ≤ BMI < 28.0 kg/m^2^), and obesity (BMI ≥ 28.0 kg/m^2^). Thus, individuals with BMI ≥ 24.0 kg/m^2^ were selected from the PBICR 2021 database as the study cohort, comprising 2,588 participants.

The inclusion criteria comprised the following: (a) participants held Chinese nationality; (b) aged ≥ 18 years; (c) BMI ≥ 24.0 kg/m^2^; (d) volunteered to participate in the research and completed a consent form; (e) could understand the content of each item in the questionnaire. The exclusion criteria included: (a) participants with unconsciousness or severe mental disorders; (b) those unwilling to cooperate or be involved in similar projects.

### 2.2 Research instruments

#### 2.2.1 General information questionnaire

This included residence, gender, age, mean monthly household income per capita, education level, marital status, occupational status, chronic conditions, quantity of children, medical payment method, current medication count (excl. supplements), smoking status, and drinking status.

#### 2.2.2 Patient health questionnaire-9 (PHQ-9)

The PHQ-9, developed by Spitzer et al. (1999), was used to measure individuals’ depression symptoms. This scale consists of 9 items, each scored 0–3 (0 = “not at all” to 3 = “nearly every day”), with a total score range of 0–27. The PHQ-9 score of ≥ 10 was used as the cut-off value for depression. In this study, Cronbach’s *α* coefficient for the PHQ-9 was 0.934.

#### 2.2.3 Eating behavior scale-short form (EBS-SF)

The EBS-SF, simplified and revised by Tayama et al. (2017) based on the Sakata Eating Behavior Scale, was used to measure individuals’ eating behavior. This scale consists of 7 items assessing: eating rhythm abnormalities, satiety perception, eating habits, body constitution cognition, meal content, emotional eating, and motivation to eat. Each item is scored on a 4-point Likert scale (1 = “strongly disagree” to 4 = “strongly agree”). Total scores range from 7 to 28, with higher scores indicating poorer eating behaviors. In this study, Cronbach’s *α* coefficient for the EBS-SF was 0.857.

#### 2.2.4 Family health scale-short form (FHS-SF)

The FHS-SF, developed by Crandall et al. (2020), was used to measure individuals’ family health. This scale consists of 10 items; each item is scored on a 5-point Likert scale ranging from 1 (“strongly disagree”) to 5 (“strongly agree”). Items 6, 9, and 10 are scored inversely. We created binary variables for each of the ten FHS-SF items. Responses of 4 or higher (indicating agreement or strong agreement) were scored as 1 and responses lower than 4 (neutrality or disagreement with the statement) received a score of 0. Total scores range from 0 to 10, with higher scores indicating a higher level of family health. The Cronbach’s α coefficient for the FHS-SF in this study was 0.841.

#### 2.2.5 Scale of perceived social support (SPSS)

The SPSS, developed by Zimet et al. (1990), was used to measure social support. The scale comprises 12 items across three subscales: Family Support, Friend Support, and Other Supports. Responses are rated on a 7-point Likert scale (1 = “strongly disagree” to 7 = “strongly agree”), with total scores ranging from 12 to 84. Higher total scores indicate better social support. The Cronbach’s α coefficient for the SPSS in this study was 0.957.

### 2.3 Statistical analysis

First, SPSS 24.0 was used to statistically describe the study population, non-normal continuous variables were represented by median (M) and quartile (IQR), and categorical variables were represented by frequency (N) and percentile (%). Second, the 9 items of PHQ-9 were used as observed variables for LPA by Mplus 8.3. Model selection was guided by the following criteria: (a) The Akaike information criterion (AIC), Bayesian information criterion (BIC), and sample size adjusted BIC (aBIC) gradually decrease with the increase of the number of categories, the smaller the value the better the model fit. (b) Lo–Mendell–Rubin likelihood ratio test (LMR) and Bootstrapped likelihood ratio test (BLRT) correspond to *p*-value < 0.05, which represents k is more appropriate than k-1 category. (c) Entropy represents the accuracy indicator to evaluate the category classification, ≥ 0.8 means the classification accuracy is > 90%. (d) Diagonal values > 0.7 in the classification probability matrix indicate reliable class assignment. In addition, to the above indicators, practical significance and interpretability are also to be considered. Third, on the basis of determining the optimal model. SPSS 24.0 was used to perform Chi-square or Kruskal-Wallis tests to compare the differences in sociodemographic variables, eating behavior, family health and social support between profiles, and statistically significant indicators were subjected to multinomial logistic regression to analyze the factors associated with depression in overweight/obese individuals when different subgroups were compared. In all analyses, a 2-tailed *p* < 0.05 was considered statistically significant.

## 3 Results

### 3.1 Common method bias test

Common method bias was assessed using two distinct strategies in this study (Podsakoff et al., 2003). (a) The results of the Harman’s single-factor test revealed that there were five factors with eigenvalues exceeding 1, and the explanatory power of the first factor was 30.18%, which was below the critical threshold of 40%. The results indicated no substantial common method bias. (b) We employed the unmeasured latent method factor (ULMF) approach to assess common method bias. All observed indicators were loaded onto both theoretical constructs and a latent method factor in the CFA model. Results showed that model fit indices (CFI = 0.885, RMSEA = 0.070, SRMR = 0.071) remained unchanged after adding the method factor, indicating no significant improvement in model fit. This suggests that common method bias was not substantial in our study.

### 3.2 Statistical description

The study included 2,588 overweight/obese participants (1,516 males [58.6%] and 1,072 females [41.4%]), with 1,887 (72.9%) urban and 701 (27.1%) rural residents. The majority were aged 18–45 years (54.9%), and most families reported a mean monthly household income per capita of 3,001–6,000 yuan (40.7%). The continuous variables in this study were expressed as M (IQR): eating behavior score = 17 (6), family health score = 38 (10), and social support score = 60 (22). As shown in Table 1.

**Table 1:** Differences in demographic and continuous variables among the latent profiles (*N* = 2,588).

Characteristics	*N* (%)	Low-level *N* = 1,355	Moderate-level *N* = 947	High-level *N* = 286	*χ*^2^/H	*p*-value
Residence						
Urban	1887 (72.9)	993	685	209	*χ^2^* = 0.259	0.878
Rural	701 (27.1)	362	262	77		
Gender						
Male	1,516 (58.6)	794	533	189	*χ*^2^ = 8.696	0.013
Female	1,072 (41.4)	561	414	97		
Age						
18–45	1,420 (54.9)	677	544	199	*χ*^2^ = 42.739	< 0.001
46–59	851 (32.9)	485	297	69		
≥60	317 (12.2)	193	106	18		
Mean monthly household income per capita (yuan)						
≤3,000	767 (27.7)	342	298	78	*χ^2^* = 13.987	0.030
3,001–6,000	1,096 (40.7)	575	369	110		
6,001–9,000	426 (15.4)	224	128	47		
≥9,001	433 (16.1)	214	152	51		
Education level						
No formal education	85 (3.3)	42	28	15	*χ*^2^ = 14.793	0.022
Junior high school and below	663 (25.6)	351	256	56		
Technical secondary/high school	474 (18.3)	269	157	48		
University and above	1,366 (52.8)	693	506	167		
Marital status						
Unmarried	511 (19.7)	204	206	101	*χ*^2^ = 66.670	< 0.001
Married	1959 (75.7)	1,086	700	173		
Divorced	61 (2.4)	31	22	8		
Widowed	57 (2.2)	34	19	4		
Quantity of children						
0	643 (24.8)	272	251	120	*χ*^2^ = 67.281	< 0.001
1	1,006 (38.9)	580	345	81		
2	759 (29.3)	413	280	66		
≥3	180 (7.0)	90	71	19		
Occupational status						
Unemployed	681 (26.3)	333	270	78	*χ*^2^ = 62.369	< 0.001
Retired	241 (9.3)	148	75	18		
Student	328 (12.7)	133	124	71		
Employed	1,338 (51.7)	741	478	119		
Chronic conditions						
Yes	760 (29.4)	369	316	75	*χ*^2^ = 11.649	0.003
No	1828 (70.6)	986	631	211		
Medical payment method						
Self - paid	422 (16.3)	162	174	86	*χ*^2^ = 64.369	< 0.001
Publicly - funded	37 (1.4)	16	15	6		
Medical insurance	2,129 (82.3)	1,177	758	194		
Current medication count (excl. supplements)						
No medication use	1906 (73.6)	1,023	676	207	*χ*^2^ = 15.415	0.017
1 medication	298 (11.5)	162	102	34		
2 medications	213 (8.2)	101	91	21		
≥3 medications	171 (6.6)	69	78	24		
Smoking status						
Non - smoker	1785 (69.0)	942	646	197	*χ*^2^ = 0.844	0.932
Ex - smoker	246 (9.5)	126	95	25		
Smoker	557 (21.5)	287	206	64		
Drinking status						
Non - drinker	1,262 (48.8)	689	447	126	*χ*^2^ = 7.600	0.107
Drank before 30 days	309 (11.9)	145	125	39		
Drank in 30 days	1,017 (39.3)	521	375	121		
Eating behavior [M(IQR)]	17 (6)	16 (5)	18 (6)	21 (4)	*H* = 335.589	< 0.001
Family health [M(IQR)]	7 (4)	8 (3)	7 (5)	5 (5)	*H* = 285.623	< 0.001
Social support [M(IQR)]	60 (21)	64 (18)	57 (19)	54 (19)	*H* = 139.942	< 0.001

### 3.3 Latent profile analysis of depression in overweight/obese individuals

Using the 9 items of PHQ-9 as explicit variables, the optimal number of potential categories was explored, fitting 1 to 5 models, with fit indices presented in Table 2. As the number of categories increased, AIC, BIC, and aBIC values showed continuous decreases, reaching minima in the 5-category model. Entropy values for categories 2–5 all exceeded 0.9, while both LMR and BLRT tests yielded *p*-values < 0.05.

**Table 2:** Potential characteristic fitting index of depression in overweight/obese individuals.

Model	AIC	BIC	aBIC	Entropy	LMR	BLRT	Category probability (%)
1	56662.967	56769.332	56712.140	–	–	–	1.00
2	43536.851	43700.893	43611.929	0.977	< 0.001	< 0.001	0.846/0.153
3	38439.479	38662.107	38541.371	0.928	< 0.001	< 0.001	0.523/0.366/0.111
4	35971.210	36252.424	36099.915	0.947	0.046	< 0.001	0.512/0.248/0.147/0.091
5	35004.788	35344.589	35160.307	0.935	0.031	< 0.001	0.04/0.15/0.41/0.31/0.09

Considering the practical significance of measurement, due to the relatively low proportion of people in certain categories of profile 4 and profile 5 models, and more analogies may disperse effective information, so the models of these two potential categories were not selected. In all categories, profile 2 model had the highest AIC, BIC and aBIC values. Combined with the model comparison results, classification accuracy and practical significance, this study considers the model with three potential categories to be the optimal model for depression in overweight/obese individuals. Additionally, Table 3 showed the attribution probability matrix for the 3 potential profiles. The average probability of attribution of each class to its corresponding potential profile ranged from 95.9 to 97.6% (all > 95%), indicating that the results of the model for the three potential profiles in this study were plausible.

**Table 3:** Average attribution probabilities of each potential profile of depression.

Classes	Profile 1	Profile 2	Profile 3
Profile1	0.971	0.029	0.000
Profile2	0.035	0.959	0.005
Profile3	0.000	0.024	0.976

**Figure 2:**
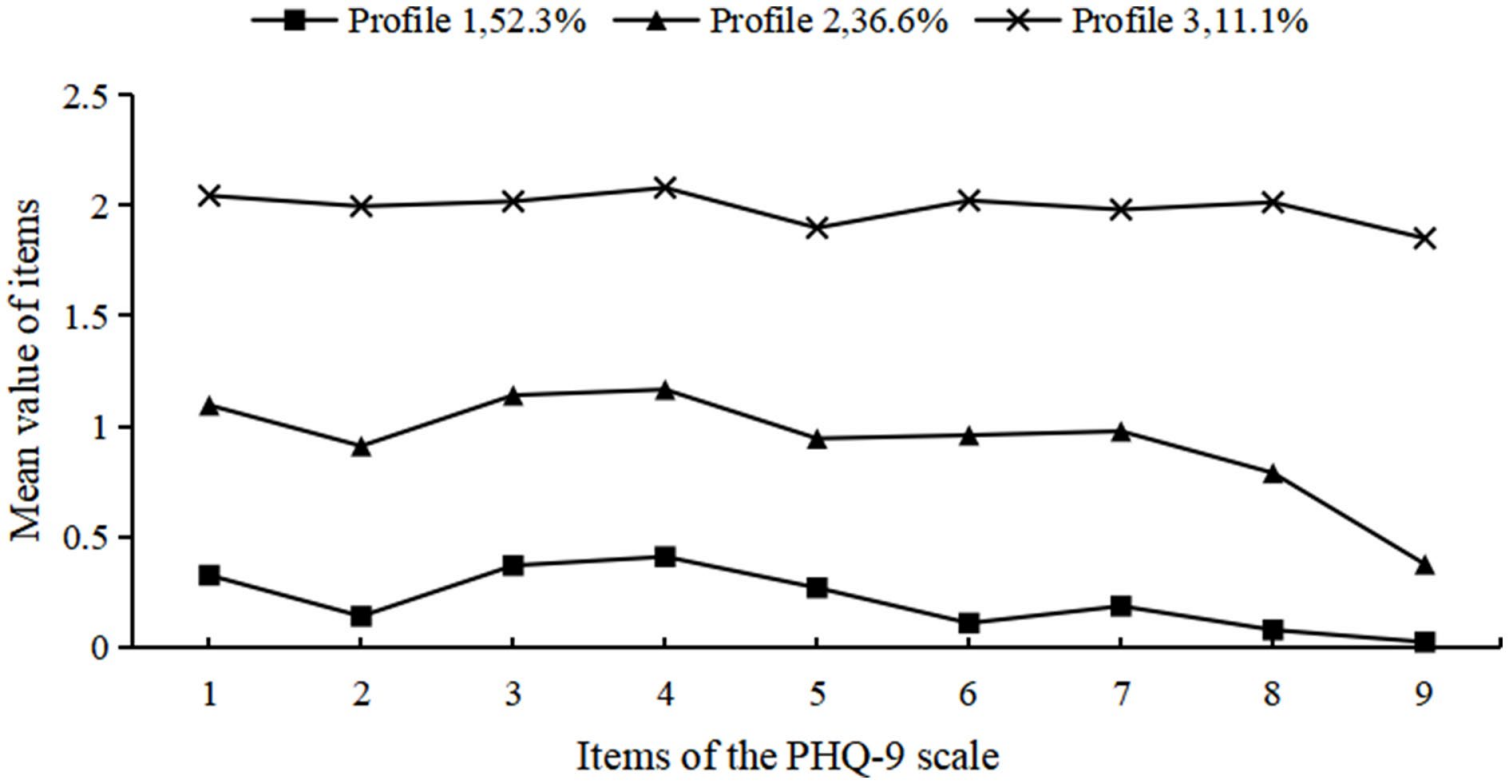
Potential profile of depression in overweight/obese individuals.

According to our study findings, we observed three distinct profiles of depression among the participants (Figure 2). The first group, comprising 1,355 individuals (52.3%), exhibited the lowest level of depression and was classified as the “low-level depression.” The second group, consisting of 947 individuals (36.6%), demonstrated moderate depression levels and was labeled the “moderate-level depression.” The third group, including 286 individuals (11.1%), displayed the most severe depression symptoms and was designated as the “high-level depression.” These findings highlight the heterogeneous nature of depression in overweight/obese individuals and emphasize the need for tailored interventions addressing each subgroup’s unique psychological characteristics.

### 3.4 Differences among latent depression profiles

Results from the Chi-square and Kruskal-Wallis tests revealed statistically significant differences (all *p* < 0.05) among the three subgroups in the following variables: gender, age, mean monthly household income per capita, education level, marital status, number of children, occupational status, chronic conditions, medical payment method, current medication count (excl. supplements), eating behavior, family health, and social support. As shown in Table 1.

### 3.5 Multivariate analysis of potential profiles of depression in overweight/obese individuals

“Low–level depression” was referenced in this study, with depression subtypes as the dependent variable (low- = 1, moderate- = 2, and high- level depression = 3), demographic and continuous variables that were statistically significant in the univariate analysis as independent variables (variable assignments are shown in Table 4), and correlates explored through multinomial logistic regression. Compared to the low-level group, high-level depression was significantly associated with age (18–45 years), current medication count (≥ 3, excl. supplements), out-of-pocket medical expenditures, higher abnormal eating behavior scores, and lower family health and social support scores (all *p* < 0.05). Similarly, moderate-level depression showed significant associations with female, age (18–45 years), having chronic conditions, current medication count (≥ 3, excl. supplements), out-of-pocket medical expenditures, higher abnormal eating behavior scores, and lower family health and social support scores (all *p* < 0.05).

**Table 4:** Case of variable assignment.

Variable	Assignment mode
Gender	Male = 1; Female = 2
Age	18–45 = 1; 46–59 = 2; ≥ 60 = 3
Mean monthly household income per capita (yuan)	≤3,000 = 1; 3,001–6,000 = 2; 6,001–9,000 = 3; ≥ 90,001 = 4
Education level	No formal education = 1; Junior high school and below = 2; Technical secondary/high school = 3; University and above = 4
Marital status	Unmarried = 1; Married = 2; Divorced = 3; Widowed = 4
Quantity of children	0 = 0; 1 = 1; 2 = 2; ≥ 3 = 3
Occupational status	Unemployed = 1; Retired = 2; Student = 3; Employed = 4
Chronic conditions	Yes = 1; No = 0
Medical payment method	Self - paid = 1; Publicly - funded = 2; Medical insurance = 3
Current medication count (excl. supplements)	No medication use = 0; 1 medication = 1; 2 medications = 2; ≥3 medications = 3
Eating behavior	Measured value
Family health	Measured value
Social support	Measured value

## 4 Discussion

### 4.1 Potential profile characteristics of depression in overweight/obese individuals

We identified three distinct depressive subtypes in overweight/obese individuals, consistent with Kong and Zhang (2023): low-, moderate-, and high-level depression groups, highlighting substantial heterogeneity in depressive phenotypes. The low-level depression group (52.3%) exhibited lower scores across all scale items compared to the other two groups, yet showed significantly higher mean scores for item 3 (‘difficulty falling asleep, waking up at night, or excessive sleep’) and item 4 (‘feeling tired or lacking energy’) relative to other items within this subgroup. This suggests that despite overall low-level depression, these individuals experience specific sleep disturbances and fatigue—likely attributable to higher prevalence of sleep apnea and other sleep disorders in overweight/obese individuals (Rodrigues et al., 2021; Chaput et al., 2023), which impair sleep quality and contribute to tiredness. Traditional binary classification (depressed vs. non-depressed) based on scale cutoffs would overlook these individuals, whereas LPA reveals that even those with low-level depression require targeted interventions, particularly for sleep and fatigue management, to prevent symptom progression (Table 5).

**Table 5:** Multivariate logistic regression analysis of different potential factors in overweight/obese individuals.

Dependent variable	Independent variable	*b*	SE	Wald *χ*^2^ value	*p*-value	OR (95% CI)
Gender (“Female” as the reference group)						
Moderate-level depression	Male	−0.204	0.095	4.654	0.031	0.815 (0.677 ~ 0.981)
	Age (“≥ 60” as the reference group)					
	18–45	0.438	0.213	4.235	0.040	1.550 (1.021 ~ 2.354)
	46–59	0.279	0.199	1.971	0.160	1.322 (0.895 ~ 1.952)
	Chronic conditions (“Yes” as the reference group)					
	No	−0.324	0.135	5.738	0.017	0.723 (0.555 ~ 0.943)
	Current medication count (excl. Supplements, “≥ 3 medications” as the reference group)					
	No medication use	−0.706	0.220	10.326	0.001	0.493 (0.321 ~ 0.759)
	1 medication	−0.671	0.226	8.786	0.003	0.511 (0.328 ~ 0.797)
	2 medications	−0.250	0.237	1.109	0.292	0.779 (0.489 ~ 1.240)
	Medical payment method (“Medical insurance” as the reference group)					
	Self - paid	0.287	0.134	4.622	0.032	1.333 (1.026 ~ 1.732)
	Publicly - funded	0.460	0.391	1.383	0.240	1.583 (0.736 ~ 3.406)
	Eating behavior	0.104	0.012	77.722	< 0.001	0.930 (0.899,0.962)
	Family health	−0.073	0.504	1.360	< 0.001	1.109 (1.084,1.135)
	Social support	−0.025	0.004	35.161	< 0.001	0.975 (0.967,0.983)
High-level depression	Age (“≥ 60” as the reference group)					
	18–45	0.990	0.387	6.527	0.011	2.691 (1.259 ~ 5.749)
	46–59	0.684	0.367	3.475	0.062	1.982 (0.65 ~ 4.070)
	Current medication count (excl. Supplements, “≥3 medications” as the reference group)					
	No medication use	−1.372	0.338	16.472	< 0.001	0.254 (0.131 ~ 0.492)
	1 medication	−0.655	0.355	3.397	0.065	0.520 (0.259 ~ 1.042)
	2 medications	−0.670	0.392	2.922	0.087	0.512 (0.238 ~ 1.103)
	Medical payment method (“Medical insurance” as the reference group)					
	Self - paid	0.788	0.189	17.382	< 0.001	2.199 (1.518 ~ 3.186)
	Publicly - funded	0.536	0.593	0.816	0.366	1.710 (0.534 ~ 5.471)
	Eating behavior	0.279	0.021	183.101	< 0.001	1.3121 (1.269 ~ 1.376)
	Family health	−0.217	0.026	71.247	< 0.001	0.805 (0.766 ~ 0.847)
	Social support	−0.019	0.007	8.127	0.004	0.981 (0.969,0.994)

The moderate-level depression group accounted for 36.6% of the sample, with significantly higher mean scores on item 3 (“difficulty falling asleep, nighttime awakening, or excessive sleep”) and item 4 (“feeling tired or lacking energy”) than other items. This pattern suggests that overweight/obese individuals in the moderate-depression subgroup also experience pronounced sleep disturbances and fatigue. Compared to the low-level depression group, all scale items in the moderate group showed higher mean scores (albeit lower than the high-level group), suggesting substantial instability—these individuals may regress to low-level depression or progress to high-level depression. Consequently, timely identification and targeted interventions become crucial to prevent transition to more severe depression.

The high-level depression group comprised 11.1% of the sample, showing the most severe depressive symptoms among the three groups. Luppino et al. (2010) reported a bidirectional association between overweight and clinical depression, with a stronger correlation observed in obesity, potentially attributable to increased social discrimination, stigmatization, and body image dissatisfaction experienced by overweight/obese individuals. Furthermore, poor dietary habits and metabolic disorders (including chronic inflammation and insulin resistance) are strongly linked to depressive severity in this population (Miller and Raison, 2016; Chen et al., 2021). Consequently, this subgroup represents the highest-risk category among the three phenotypes, requiring intensive interventions to understand their psychological state, alleviate emotional distress, and enhance quality of life.

### 4.2 Associated factors of potential profiles of depression in overweight/obese individuals

#### 4.2.1 Individual characteristics

##### 4.2.1.1 Gender

In our study, female participants demonstrated a significantly higher likelihood of being classified into the moderate-level depression group. This finding aligns with existing literature, as Johann and Ehlert (2022) documented that women exhibit approximately twice the risk of developing depression compared to men throughout the lifespan. Further supporting this observation, Tang and Zhang (2022) demonstrated that females generally display greater sensitivity to social evaluation and stronger need for social affirmation than males, coupled with reduced resilience to negative feedback. These psychological characteristics may explain why overweight/obese women tend to experience heightened body image concerns and greater distress in response to negative social evaluations, consequently contributing to their elevated depressive symptoms.

##### 4.2.1.2 Chronic conditions

In this study, individuals with chronic conditions were more likely to exhibit moderate-level depression. This finding is consistent with previous study (Liu et al., 2023). Zhou et al. (2023) indicated that chronic conditions, characterized by prolonged incurability and necessitating lifelong treatment, impose not only considerable psychological and physiological burdens on patients but also substantial economic strains and productivity losses for families, thereby increasing susceptibility to depression. For overweight/obese individuals with chronic conditions, the dual challenge of managing both body weight and chronic illnesses likely intensifies physical and mental stress, consequently elevating depressive symptoms.

##### 4.2.1.3 Age

This study found that overweight/obese individuals aged 18–45 demonstrated elevated depressive symptoms. As reported by Strand et al. (2021), BMI serves as the strongest predictor of body image disparities in adults. This age group, predominantly comprising university students and working professionals, faces heightened career and familial demands, resulting in increased self-image concerns (Aruna et al., 2024). Overweight/obesity can induce body dissatisfaction and weight-related stigma, which negatively impact self-esteem and psychological well-being (Timkova et al., 2024). Furthermore, these individuals frequently experience weight-based discrimination, teasing, and bullying. Such prejudice, prevalent across multiple social domains including workplaces, educational settings, and interpersonal relationships, significantly contributes to various psychological comorbidities, particularly depression, anxiety, and social isolation (Puhl and King, 2013).

##### 4.2.1.4 Current medication count (excl. supplements)

This study indicated that overweight/obese individuals prescribed ≥3 medications exhibited more severe depressive symptoms. This observation aligns with previous research. Assari and Bazargan (2019) reported that polypharmacy is associated with elevated psychological distress, while Kyler et al. (2023) showed that nearly half of obese individuals receive multiple medication regimens. This phenomenon results from the high prevalence of obesity-related comorbidities (e.g., diabetes, dyslipidemia, hypertension and metabolic syndrome) (Ramón-Arbués et al., 2019), which necessitate polypharmacy. The increased number of medications, combined with side effects, drug interactions, and financial burdens—can exacerbate psychological stress and predispose to depression (Assari et al., 2019).

#### 4.2.2 Individual behaviors level

##### 4.2.2.1 Eating behavior

We found that overweight/obese individuals with abnormal eating behaviors had more severe depressive symptoms, consistent with previous studies (Masheb and Grilo, 2006). Emotional eating, binge eating, and food addiction are common coping ways for many overweight/obese people to cope with negative emotions such as stress and anxiety (Isnard et al., 2003; Esen Öksüzoğlu et al., 2024). Fulton et al. (2022) demonstrated that the mental consequences of obesity stem from poor diet, lack of exercise, and visceral fat accumulation, and the resulting metabolic and vascular dysfunctions, including inflammation, insulin and leptin resistance, and hypertension, have become major risks for the development of depression and anxiety. For example, high-fat and high-sugar diets can cause chronic low-grade inflammation, and proinflammatory cytokines (TNF-*α*, IL-6) are closely related to the occurrence of depression (Miller and Raison, 2016). Dietary modification and physical activity represent fundamental strategies for establishing healthy eating habits in overweight/obese populations. Empirical evidence suggests the Mediterranean diet is particularly suitable for this demographic (Khandelwal, 2020). Furthermore, yoga practice, aerobic exercise, and regular sleep patterns have been shown to enhance stress management, thereby facilitating healthier dietary behaviors (Watts et al., 2018; Khandelwal, 2020). Regarding psychological support, Hanson et al. (2019) demonstrated that mindfulness-based eating interventions effectively improve maladaptive eating behaviors in obese individuals.

#### 4.2.3 Interpersonal networks level

##### 4.2.3.1 Social support

Our data demonstrated that higher social support was associated with lower depressive symptoms in overweight/obese individuals, consistent with the findings of Timkova et al. (2024). Social support, as a critical external resource available to individuals, plays a positive role in enhancing health-promoting behaviors, improving self-worth, and reducing anxiety and depression (Langford et al., 1997). For overweight/obese individuals, establishing specialized health management institutions, regular supervision and follow-up by healthcare professionals, and creating healthy workplaces can help them feel social acceptance and support. Additionally, support from peers and family is also important social support resources. Adequate social support can not only facilitate active weight management but also enhance their ability to cope with difficulties and negative emotions.

#### 4.2.4 Community level

##### 4.2.4.1 Family health

We found that better family health was associated with less severe depressive symptoms in overweight/obese individuals. Hao et al. (2023) reported that decreased family health functioning directly influences individual depressive symptoms. Healthy family social and emotional processes can promote health resilience and are associated with better mental health, physical health, and overall well-being (e.g., reduced depression, hypertension and chronic pain) (Chew et al., 2018). Good family health implies adequate internal emotional communication among family members, accessible health resources, and external social support, and emphasizes the adoption of healthy lifestyles to promote individual physical and mental health (Mei et al., 2022). Thus, optimal family health not only helps overweight/obese individuals adopt healthy eating habits and maintains regular routines but also enables effective coping with negative emotions and protects psychological well-being when facing stigma or stress. These findings highlight the importance of addressing family health functioning in overweight/obese individuals, suggesting that promoting family health is a key strategy to improve their mental health outcomes.

#### 4.2.5 Policy level

##### 4.2.5.1 Medical payment method

The results demonstrated that overweight/obese individuals with out-of-pocket medical expenses exhibited more severe depressive symptoms. Studies have shown that negative societal stereotypes about obesity lead to unfair treatment of obese individuals in job seeking and salary levels, which may limit their economic income (Judge and Cable, 2011; Campos-Vazquez and Gonzalez, 2020). Out-of-pocket medical expenses further exacerbate their financial strain and psychological distress, thus exacerbating depressive symptoms (Zhou et al., 2023). To address this issue, public health policies should prioritize implementing medical expense subsidies for overweight/obese populations while enforcing anti-discrimination legislation in workplaces to ensure equitable employment opportunities and income levels.

##### 4.2.5.2 Other macro-level policies

It is noteworthy that the “Healthy Diet Initiative” and “National Fitness Program” advocated in the “Healthy China 2030” action plan,^2^ along with the “whole-population, life-cycle coverage and precision weight management” proposed in the “National Weight Management Year” implementation plan,^3^ collectively demonstrate China’s policy-level efforts toward scientific weight management and chronic disease control. These initiatives provide a distinctive Chinese public health solution for advancing the United Nations Sustainable Development Goal 3 (SDG-3) - “Ensure healthy lives and promote well-being for all at all ages”.

##### 4.2.5.3 Strengths and limitations

In this study, First, employing LPA, the study identified distinct depression subtypes among overweight/obese individuals. Second, grounded in the SEM, the study systematically explores multi-level associated factors of depression subtypes, transcending traditional single-dimensional analyses. Third, the nationwide cross-sectional study design incorporated a large, nationally representative sample of 2,588 Chinese participants, enhancing the generalizability of the findings. Collectively, this research provides evidence-based insights for formulating targeted multi-level interventions among overweight/obese individuals.

Several potential limitations of this study should be acknowledged. Firstly, due to the cross-sectional study design, we cannot infer causal implications. Secondly, as a result of the self-reported information and the self-assessed scales in the study, reporting bias may exist. Finally, although the study examined multiple factors related to depression subtypes among overweight/obese individuals within the SEM framework, only one policy-environmental factor (medical payment method) was considered in this study. Future studies should enrich the policy-environmental dimension of the model by incorporating specific contextual variables, such as regional mental health policies or urban infrastructure.

## 5 Conclusion

Depression in overweight/obese individuals was categorized into three subtypes: low-, moderate-, and high-level depression groups. An in-depth understanding of these factors across five SEM levels helped formulate more targeted and multidimensional intervention strategies. Significant differences were observed across these groups in age, gender, chronic conditions, medication use, eating behavior, family health, social support, and medical payment method. Therefore, interventions for high-risk populations should not only address weight management and physical health improvement but also prioritize mental health.

## Data Availability

The datasets presented in this study can be found in online repositories. The names of the repository/repositories and accession number(s) can be found in the article/supplementary material.

## Footnotes

^1^ https://www.x-mol.com/groups/pbicr

^2^ https://www.gov.cn/xinwen/2019-07/15/content_5409694.htm

^3^ https://www.gov.cn/zhengce/zhengceku/202406/content_6959543.htm
